# Alpha-Synuclein Continues to Enhance SNARE-Dependent Vesicle Docking at Exorbitant Concentrations

**DOI:** 10.3389/fnins.2019.00216

**Published:** 2019-03-21

**Authors:** Brenden J. D. Hawk, Ryan Khounlo, Yeon-Kyun Shin

**Affiliations:** Roy J. Carver Department of Biochemistry, Biophysics and Molecular Biology, Iowa State University, Ames, IA, United States

**Keywords:** SNARE, alpha-synuclein, single-molecule fluorescence, membrane fusion, supported bilayer, total internal reflectance fluorescence

## Abstract

Recently, Parkinson’s disease-associated α-synuclein (αS) has emerged as an important regulator for SNARE-dependent vesicle fusion. However, it is controversial if excessive accumulation of αS, even in the absence of aggregation, impairs neurotransmission. Here we use a single vesicle fusion assay with ms time resolution capable of dissecting the impact of αS on each step of membrane fusion. Unlike the previous results from various *in vitro*, cellular, and *in vivo* studies, we find that non-aggregated αS promotes vesicle merger even at exorbitant concentrations. The enhancement has been seen as much as 13 fold. Delving into the kinetics of the intermediate states for vesicle fusion reveals that αS stimulates vesicle docking without altering the dynamics of bilayer merger (lipid mixing). However, minute amounts of soluble aggregated species abolish SNARE-dependent bilayer merger completely. Thus, the results show that excessive accumulation of non-aggregated αS may not be toxic for neurotransmitter release.

## Introduction

Neurotransmitter release is the foundation of thought in the central nervous system. The brain is comprised of networks of neurons joined together via synapses. Communication between these neurons is mediated by vesicle fusion releasing neurotransmitters at the synapses. Defects in the regulation of neurotransmission are believed to be the primary cause for many neurological diseases. For example, the main cause for Schizophrenia is thought to be over-release of dopamine, while for Epilepsy it may be the under-release of adenosine and glycine (Boison et al., 2012). The key to a better understanding of neurological diseases may lie in a more complete understanding of the molecular machine responsible for synaptic vesicle fusion.

The SNARE (Soluble *N*-ethylmaleimide-sensitive factor Attachment Protein Receptor) proteins are believed to mediate synaptic vesicle fusion (Söllner et al., 1993; Weber et al., 1998; Jahn and Scheller, 2006). Vesicle-associated (v-) SNARE forms a helical coiled coil with the target plasma membrane (t-) SNARE to drive membrane fusion (Poirier et al., 1998; Sutton et al., 1998). Neurotransmitters are released from the vesicle through the fusion pore to the synaptic cleft (Breckenridge and Almers, 1987). Action potential-evoked vesicle fusion events are synchronized and happen quickly (in less than a millisecond) (Sabatini and Regehr, 1987). The synchronous release is achieved through an exquisite regulation of SNARE complex formation by a host of regulatory proteins including the Ca^2+^-sensor, synaptotagmin-1 (Fernandez et al., 2001; Chapman, 2002; Lee et al., 2010).

Alpha-synuclein (αS) has long been a protein of great interest because of its pathological aggregation in neuronal plaque and Lewy bodies, which are associated with Parkinson’s Disease and Lewy Body Dementia, respectively (Hardy and Gwinn-Hardy, 1998; Shimohama et al., 1998; Lee and Trojanowski, 2006; Spillantini et al., 2010). Despite its medical significance, the normal function of αS has been elusive. αS is a 14 kDa soluble protein that is abundantly present in the neuron (several μM), particularly in the presynapse (Maroteaux et al., 1988; George et al., 1995). Not surprisingly, αS has been found to play roles in various aspects of vesicle recycling (Larsen et al., 1988; Nemani et al., 1988; Yavich et al., 1988; Scott and Roy, 2012). Recently, αS has emerged as an important regulator for SNARE-dependent vesicle fusion.

Sudhof and coworkers discovered that αS promotes SNARE complex formation via its interaction with v-SNARE VAMP2 (Burré et al., 2010). Consistent with these results, Lou et al. (2010) found in an *in vitro* study that αS promotes vesicle docking. However, the proposed positive role of αS in SNARE-dependent membrane fusion appears to be at odds with the results from nearly all overexpression studies. The overexpression studies show that αS leads to the impairment of neurotransmission (Larsen et al., 1988; Nemani et al., 1988; Lundblad et al., 2010; Phan et al., 2017). Although it was shown that overexpressed αS disturbs vesicle pools (Nemani et al., 1988), its direct interference with the SNARE complex formation cannot be ruled out. Furthermore, several *in vitro* studies have shown that excessive αS severely inhibits SNARE-dependent membrane fusion (DeWitt and Rhoades, 2013; Lai et al., 2013). Thus, the results raise the question if the αS function is acutely concentration-dependent and, furthermore, if excessive αS is a cause of the impaired neurotransmission. If true, this might have serious medical implications. Similarly, for Alzheimer’s disease, excessive but non-aggregated amyloid-β has been suspected for the impaired neurotransmission and early symptoms of the disease (Bergamaschini et al., 1999; Lai and McLaurin, 2010).

To sort out the discrepancy among the results, we used an *in vitro* single-vesicle-to-supported bilayer merger assay (Kim and Shin, 2010), in which, unlike the cellular environment, the precise variation of αS concentration is possible. Moreover, this assay provides the opportunity to dissect the impact of αS on individual vesicle fusion steps such as vesicle docking and bilayer merger along the membrane fusion pathway. The assay has proven effective in analyzing single bilayer merger events in a natural, millisecond timescale between vesicle and a supported-bilayer (Kim and Shin, 2010).

Using this assay, we have found that αS enhances vesicle docking by a factor of 10 at an excessive 20 μM concentration, which is as much as 4 times higher than normal cellular levels. These results contradict the notion that excessive αS impairs vesicle fusion and neurotransmission, which is previously supported by overexpression as well as the *in vitro* studies. Furthermore, the detailed analysis reveals that the effect of αS on SNARE-dependent membrane fusion is largely on vesicle docking with no significant changes in the dynamics of bilayer merger.

## Materials and Methods

### Plasmid Constructs and Site-Directed Mutagenesis

DNA sequences encoding syntaxin-1A (amino acids 1-288 with three native cysteines replaced by alanines), VAMP2 (amino acids 1-116 with C103 replaced by alanine), SNAP-25 (amino acids 1-206 with four native cysteines replaced by alanines), and αS (amino acids 1-140) were inserted into a pGEX-KG vector as N-terminal glutathione S-transferase (GST) fusion proteins. DNA sequences were confirmed by the Iowa State University DNA sequencing facility.

### Protein Purification

All N-terminal GST recombinant neuronal SNARE proteins and αS were expressed in *Escherichia coli* BL21 (DE3) cells. SNARE proteins were purified in the same manner as previously detailed (Khounlo et al., 2017).

The αS was grown at 37°C in LB (Luria–Bertani) medium with 100 μg/mL ampicillin until the absorbance at 600 nm reached 0.6–0.8. The cells were induced to express overnight by adding IPTG (isopropyl β-D-thiogalactopyranoside, 0.3 mM final concentration) at 16°C. Proteins were purified using glutathione-agarose chromatography. Cell pellets were resuspended in 15 ml of high salt PBS (HSPBS) (497 mM NaCl, 2.7 mM KCl, 10 mM Na2HPO4, 1.8 mM KH2PO4, pH 6.5), with final concentrations of 1 mM AEBSF [4-(2-aminoethyl)benzenesulfonyl fluoride], 2 mM DTT (dithiothreitol), and 20% *N*-lauroylsarcosine. Cells were lysed by homogenization and centrifuged at 25,000 ×*g* for 30 min at 4°C. The supernatant was mixed with 1 ml of glutathione-agarose beads in HSPBS by nutation at 4°C for 2 h. After washing the protein with HSPBS, the protein was cleaved by thrombin (30 U) at 4°C overnight. The protein was eluted, concentrated to ∼1 ml, and then loaded on to an AKTA FPLC with a GE Superdex Increase 200 10/300 column for size-exclusion chromatography exchanging αS into PBS (137 mM NaCl, 2.7 mM KCl, 10 mM Na2HPO4, 1.8 mM KH2PO4, pH 7.4). Samples from individual fractions were run on a 15% SDS-PAGE gel to identify fractions that lacked high molecular weight bands (Supplementary Figure S1). These fractions were then combined, concentrated to ∼1 mL, and subjected to the FPLC again. Fractions were again checked for any high molecular weight bands. Those without were combined, concentrated, had glycerol added to a final concentration of 15%, and then stored at -80 C (Supplementary Figure S1). αS was used within 2 weeks of purification. Aggregation into high molecular weight bands within this time frame was negligible (Supplementary Figure S2).

### Western Blotting

Alpha-synuclein was run on a 12% SDS-PAGE. SDS was rinsed from the gel by microwaving the gel in diH_2_O (deionized water) for 40 s three times with each time exchanging the diH_2_O. An Invitrogen iBlot^TM^ two Dry Blotting System was used to transfer to a PVDF membrane. The membrane was then blocked overnight with 5% (w/v) non-fat dry milk in TBST (20 mM Tris–HCL, pH 7.4, 137 mM NaCl, 2.7 mM NaCl, 0.1% Tween-20) at 4 C. The membrane was rinsed with TBST twice, then incubated in 5% (w/v) non-fat dry milk in TBST with 1 μg/mL alpha Synuclein Monoclonal Antibody (Syn 211) (Invitrogen #32-8100) for 2 h at room temperature. Then, the membrane was rinsed three times with TBST and incubated with 5% (w/v) non-fat dry milk in TBST with Goat Anti-Mouse IgG (H + L)-HRP Conjugate (Bio-RAD #1706516) for 45 min at room temperature. The membrane was then washed with TBST three times and reacted with Pierce^®^ ECL Western Blotting Substrate (Thermo Fisher 32106) for 1 min. The western blot was visualized with a ChemiDOC system (Bio-Rad).

### Membrane Reconstitution

The t-bilayer was made using a mixture of POPC (1-palmitoyl-2-dioleoyl-sn-glycero-3-phosphatidylcholine), DOPS (1,2-dioleoyl-sn-glycero-3-phosphatidylserine), PIP_2_ (phospha-tidylinositol 4,5-bisphosphate from porcine brain), and PEG2000 (1,2-dipalmitoyl-sn-glycero-3-phosphoethanolamine-N-[methoxy(polyethylene glycol)-2000]) in chloroform at a molar ratio of 78:15:2:5. The v-lipid was made using a mixture of POPC, DOPS, cholesterol, and DiI (1, 1′-Dioctadecyl-3, 3, 3′, 3′-tetramethylindocarbocyanine perchlorate) in chloroform at a molar ratio of 54:5:40:1. Both lipid mixtures were first dried under an air stream, then dried in a vacuum overnight. The t-bilayer lipid was resuspended in HEPES (25 mM HEPES/KOH, 150 mM KCl, pH 7.4) + 1% OG (Octyl-beta-Glucoside). The v-lipid was resuspended in HEPES and then underwent 10 flash freeze-thaw cycles moving from liquid nitrogen to boiling water. Protein-free large unilamellar vesicles (∼100 nm in diameter) were prepared by extrusion through polycarbonate filters making v-liposomes.

For the t-bilayer syntaxin-1A and SNAP-25 in a molar ratio of 1:1.5, were premixed, and the mixture was left at room temperature for 0.5 h to form the t-binary complex before the reconstitution. The t-bilayer lipid was added to the t-binary complex so that the lipid to t-binary complex ratio was 2000:1. Then, the liposome/protein mixture was diluted by adding three times the volume of the protein lipid mixture of HEPES. To generate functional v-vesicles, v-liposomes were mixed with the VAMP2 at a lipid to VAMP2 ratio of 200:1. Then the liposome/protein mixture was diluted by adding three times the volume of the protein lipid mixture using HEPES. Both liposome/protein mixtures were dialyzed overnight at 4°C in 2 L of HEPES containing Biobeads SM-2 Resin.

### Vesicle-to-Supported Bilayer Merger TIRF Assay

A quartz slide and glass cover slip were subjected to piranha cleaning by boiling them in a 1:1 mixture of concentrated sulfuric acid and 30% hydrogen peroxide for 15 min. The slides were rinsed with diH_2_O and placed in a cleaning sonicator for 30 min to remove residual acid. The slides were dried and prepared with the double-sided tape and the cover slip to generate several separate microfluidic chambers. The chambers were then filled with t-bilayer that was prepared from the overnight dialysis. The t-bilayer was allowed to form on the quartz surface for 1 h at room temperature. The excess liposomes/protein mixture was washed out with HEPES and replaced with an indicated concentration of αS in HEPES. The αS was incubated with t-bilayer for 1 h while heating at 37°C.

**FIGURE 1:**
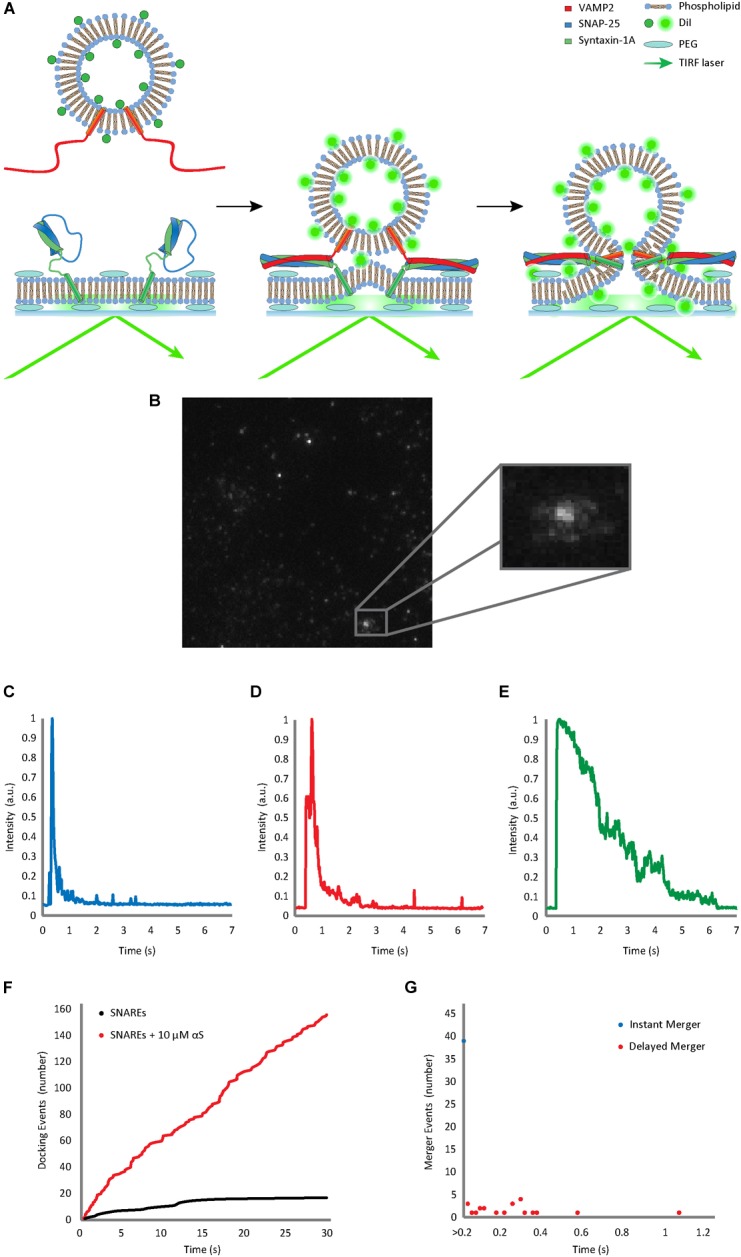
Single vesicle-to-supported bilayer merger assay distinguishes vesicle docking from bilayer merger in real time. **(A)** Schematic diagram of the single vesicle-to-supported bilayer merger assay. V-vesicles are labeled with 1 mol % lipid-dye DiI. The t-bilayer is stabilized by a PEG (polyethylene glycol) cushion between the bilayer and the quartz slide. When v-vesicles dock onto the t-bilayer through SNARE zippering, the TIRF laser excites the fluorescent dye allowing the individual v-vesicle to be visualized. **(B)** Lipid diffusion on the bilayer surface due to bilayer merger. The large box contains a raw image of the docked and merging vesicles. The small box contains a zoomed image of lipid diffusion event. **(C)** Representative trace of the instant merger. **(D)** Representative trace of the delayed merger. **(E)** Representative trace of docking only. The fluorescence decay due to photo-bleaching is at least 5 times slower than the decrease due to lipid diffusion. Further classification of instant merger, delayed merger, and docking only events are defined in the Materials and Methods section. **(F)** Representative cumulative plot of docking events occurring within a single viewing area for 30 s. The black line indicates cumulative docking events without αS. The red line indicates those with twice purified αS at 10 μM. **(G)** Representative scatter plot of the delay from docking to bilayer merger. Bilayer merger events that occur within the first 20 ms after docking are classified as instant merger, whereas merger events that occur later are classified as delayed merger.

The quartz slide with the microfluidic chambers was then placed on the imaging stand of our microscope, which regulated the temperature of the slide to 37 C. Imaging oil was put on the prism of our prism-type total internal reflection fluorescence (TIRF) microscope, and then the prism was lowered onto the quartz slide. The incident angle of the exciting laser (532 nm) was adjusted and we initiated real-time movie acquisition with an imaging area of ∼55 ×∼110 μm using 20 ms time resolution. We then gently injected the v-vesicles from dialysis with an indicated concentration of αS into the microfluidic chamber. The injection pump was promptly stopped after injection to prevent vesicle merger events from the flow effect (Figure 1A). We collected 60 s movies for each microfluidic chamber and analyzed the final 30 s for docking and merger events using our custom-built analysis software.

### Data Analysis

Using our home-made analysis software written in MATLAB^®^ 2014 (b), we monitored the fluorescence intensity of DiI from the v-vesicles in order to determine docking and subsequent merger events. Each recording was analyzed frame by frame based on both visual determination (Figure 1B) and fluorescence trace pattern analysis (Figure 1C–E). Both criteria had to be met for an event to be counted. All three events are initiated by both a vesicle becoming immobilized on the surface and a sharp increase in the fluorescence. Instant merger events were then defined as lipid diffusion happening on the very next frame as well as an immediate sharp fluorescence decrease (Figure 1D). Delayed merger events were defined by lipid diffusion being observed several frames after docking, with a secondary fluorescence spike upon lipid merger, followed by an immediate, sharp decrease (Figure 1E). Docking only events did not show lipid diffusion before the fluorescence intensity faded gradually over time due to photo-bleaching (Figure 1F).

From the analysis, we counted individual immobilized v-vesicles, vesicles showing lipid merger, and the delay time between docking and lipid merger within a movie clip. At least three independent recordings were analyzed to obtain the statistical significance for each data set.

### Exponential Curve Fitting

All membrane merging data was combined for each concentration of αS. The membrane merging events were cumulatively plotted by binning events by the maximal delay. For example, if the event was seen to fuse in a single frame, then it was binned in the 0–20 ms bin and plotted as an event at 20 ms. MATLAB^®^ 2014 (b) curve fitting toolbox^TM^ 3.5 was used to fit *F*(x) = A^∗^(1-exp((-x)/B))+C^∗^(1-exp((-x)/D)+E to the cumulative plot allowing A, B, C, D, and E to be optimized using Non-linear Least Squares with Bisquare for robustness. Standard deviations were calculated by taking all events for a concentration of αS and assigning them randomly to a subgroup, fitting each subgroup, and comparing their best fitting parameters to the global fit to the total data.

## Results

### Single Vesicle-to-Supported Bilayer Merger Assay

To investigate the effect of αS on SNARE-dependent vesicle fusion, we analyzed the merger of single v-SNARE VAMP2-reconstituted vesicles (v-vesicles) to the supported bilayer reconstituted with t-SNAREs syntaxin-1A and SNAP-25 (t-bilayer) with TIRF microscopy (Figure 1A). Similar experimental platforms have been previously used by several groups to investigate SNARE-dependent membrane fusion (Fix et al., 2004; Karatekin et al., 2010; Liu et al., 2010; Kiessling et al., 2017). We adopted this method instead of the vesicle-to-vesicle fusion method (Yoon et al., 2006; Lou et al., 2010) because the supported bilayer better mimics the planar geometry of the plasma membrane. The planar t-bilayer rests on polyethylene glycol (PEG) supports to maintain the membrane fluidity (Karatekin et al., 2010; Kim and Shin, 2010). The v-vesicles are doped with 1 mol % lipid-dye DiI as a reporter for the diffusion of phospholipid molecules from a v-vesicle onto the planar bilayer upon the merging of lipid bilayers.

Immediately after v-vesicles were flowed over the supported bilayer in the microfluidic chambers, vesicle docking and bilayer merger events were filmed every 20 ms for 30 s. Individual docking and subsequent bilayer merger events could be resolved from each other. When a vesicle is tethered to the surface, docking caused a sharp increase in fluorescence, but when the bilayers merged, the lipids diffused in the two-dimensional (2-D) surface creating a steep decline in fluorescence at the centroid (Figure 1B–D). The cumulative number of vesicle docking events (Figure 1F) and the delay between docking and bilayer merger were recorded for further analysis of the kinetics (Figure 1G). We found that about 40–60% of docked vesicles exhibit bilayer merger (Figure 1F–G). Of those events, nearly 70% showed instant bilayer merger (within the first 20 ms), while 30% were scattered over the 30 s recording time (Figure 1G).

### αS Enhances Vesicle Docking in all Concentrations Studied up to 20 μM

**FIGURE 2:**
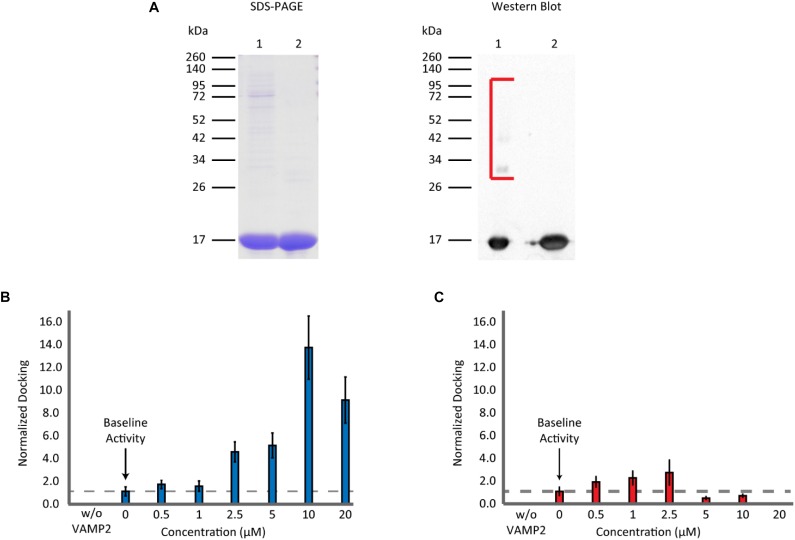
Non-aggregated αS enhances vesicle docking activity even at excessive concentrations. **(A)** SDS-PAGE and western blot analysis of purified recombinant αS. A 12% SDS-PAGE gel containing αS through the two rounds of gel filtration: Lane 1 contains αS after the 1st round of gel filtration. Lane 2 contains αS after the 2nd round of gel filtration (left panel). A western blot of αS through the two rounds of gel filtration (right panel). The lane order is the same as SDS-PAGE. Bracket encloses higher molecular weight αS bands observed only in the sample from a single round of gel filtration. **(B)** Analysis of vesicle docking with twice purified αS (lane 2 in A). The number of docking events for each αS concentration is normalized relative to that without αS. The dashed line represents the vesicle docking activity of SNAREs without αS. Studies without VAMP2 were done by flowing v-liposomes without VAMP2 reconstituted in them along with 10 μM αS over the supported t-bilayer. No events were observed. In total 3,148 docking events from 82 independent measurements are analyzed. **(C)** Analysis of vesicle docking with one-time purified αS (lane 1 in A). The number of docking events from each αS concentration is normalized with respect to those without αS. The dashed line represents the docking activity of SNAREs without αS. Studies without VAMP2 were done in the same way as in B. A total of 6,249 vesicle docking events from 65 independent measurements are analyzed.

Recombinant αS was expressed in *E. coli* as a GST-fusion protein and it was purified with gel-filtration chromatography after cleavage from the GST-tag. SDS-PAGE gels and western blots showed that the protein still had trace amounts of high molecular weight bands after one round of gel filtration (Figure 2A). Thus, the sample was subjected to a subsequent round of gel filtration to attain a higher purity. When this highly purified, twice filtered αS was included in the single-vesicle assay, it enhanced SNARE-dependent vesicle docking events in a concentration-dependent manner (Figure 2B). It promoted vesicle docking dramatically with as much as a 13 fold at 10 μM and 10 fold even at 20 μM. The results were surprising and appear to contradict previous results from overexpression studies (Nemani et al., 1988) and those from several *in vitro* studies (DeWitt and Rhoades, 2013; Lai et al., 2013), where 20 μM αS almost completely abolished bilayer merger and subsequent fusion. We tested the SNARE dependence of the αS enhancement by removing the SNARE component on the vesicles (VAMP2). Without VAMP2 membrane docking and merger was abolished. 10 μM αS did not generate any events in the absence of VAMP2 demonstrating the SNARE dependence of αS enhancement (Figure 2B).

We wondered if the discrepancy between the present and previous results stemmed from the high molecular weight bands in under-purified samples. To test this, we used αS from the 1st gel filtration, containing small amounts of high molecular weight bands, in our bilayer merger assay. We observed a small enhancement in vesicle docking at lower concentrations. However, there was a significant inhibition of vesicle docking at αS concentrations greater than 5 μM with vesicle docking and bilayer merger activity almost non-observable at 20 μM (Figure 2C). This concentration-dependent variation is reminiscent of a previous *in vitro* study by Lou et al. (2010) using the single vesicle-to-vesicle fusion assay. The drastic effect from the small amount of high molecular weight bands from a single round of gel filtration could also explain why the enhancement of the highly purified αS plateaued after 10 μM. A minute amount of the high molecular weight bands could still be present in the highly purified αS, and thus negate the continued enhancement of the non-aggregated αS.

**FIGURE 3:**
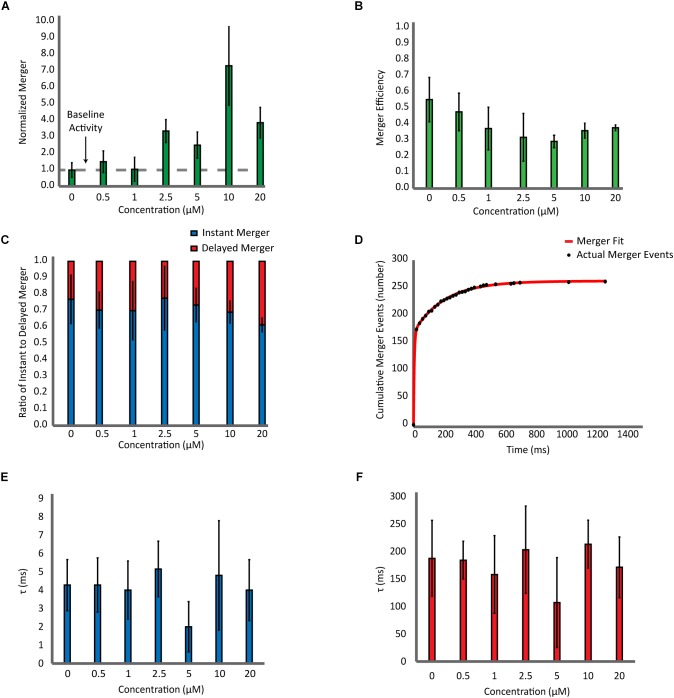
Effect of αS on SNARE-dependent membrane fusion is mainly on docking with little influence on the dynamics of bilayer merger. **(A)** Analysis of the bilayer merger dynamics with twice purified αS. The number of merger events at various αS concentrations are normalized with respect to events without αS. The dashed line represents the membrane merger activity of SNAREs without αS. **(B)** Analysis of the bilayer merger efficiency of twice purified αS. The efficiency is calculated as the number of merger events divided by the number of docking events. **(C)** Instant merger vs. delayed merger. Instant merger events display lipid-diffusion in under 20 ms and delayed merger events are any that take longer than 20 ms. **(D)** Representative cumulative plot of bilayer merger events with twice purified αS. The red line is the double exponential fit to the data of merger events. **(E)** Plot of time constants for instant merger events. **(F)** Plot of time constants for delayed merger events. The total of 1,247 merger events from 82 independent recording were included in the fitting analysis.

Next, with the highly purified αS, we carried out a detailed analysis which includes the number of bilayer merger events at various αS concentrations (Figure 3A), the percentage of docked vesicles that merged (Figure 3B), the percentage of instant merging vesicles vs. delayed merging vesicles (Figure 3C), and the kinetics of the time delay from docking until bilayer merger (Figure 3D–F). Upon analysis of the number of bilayer merger events with and without αS, we found that increasing the amount of αS led to an increase in the amount of bilayer merger, even at excessive 20 μM concentrations (Figure 3A). In order to dissect the specific role of αS, we investigated the bilayer merger efficiency. The merger efficiency (Figure 3B) shows that although there is a small decrease of the efficiency as αS increases, it seems that the variation is overall within experimental errors. Thus, the data shows that αS does not significantly affect the bilayer merger efficiency, revealing that the increase in bilayer merger events with αS is mainly due to its dramatic upstream enhancement of vesicle docking.

Next, we analyzed the time delay from docking until bilayer merger (Figure 3C). For all αS concentrations studied, nearly 70% of all bilayer mergers happen within the first 20 ms after docking. The rest (30%) are spread over the 30 s recording time. We did not observe any significant changes on these results with αS. Further on, we fit the cumulative merger events with a double exponential to find the first-order time constants for both instant and delayed merger events (Figure 3D). The time constant for instant merger varies between 2 and 5 ms (Figure 3E) while the time constant for delayed merger varies between 100 and 200 ms (Figure 3F). Neither of the time constants changes significantly with αS. Thus, the results show that αS has little effect on the time delay from docking to bilayer merger.

### Vesicles Are Not Clustered Upon Docking Onto the t-bilayer Surface in the Presence of αS

**FIGURE 4:**
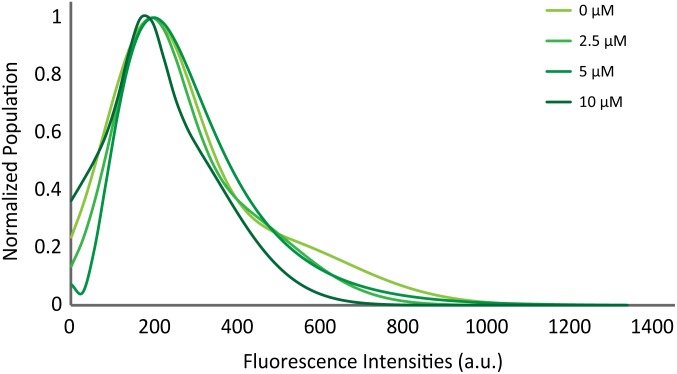
αS does not promote clustering of vesicles on the supported bilayer. Distribution of initial fluorescence intensities of individual docking events at various concentrations of twice purified αS. The maximum population for each concentration is normalized to 1 for comparison. The initial intensities of 2,194 docking events from 58 independent recordings are analyzed.

Previously, Diao et al. (2013) showed with a single vesicle-to-vesicle assay that non-aggregated αS induces vesicle clustering and argued that vesicle clustering is the cause of the enhancement of SNARE complex formation by αS. To examine if vesicles are clustered upon docking on the supported bilayer, we measured the initial fluorescence intensities of all docking events. An increase in the intensity would correlate to an increase in the number of vesicles involved in a docking event. The intensity vs. population plot shows that the αS does not shift or broaden the intensity distribution (Figure 4), indicating that αS-induced vesicle clustering does not play a role in the enhancement of vesicle docking.

## Discussion

There have been many attempts to elucidate the physiological role of normal, non-aggregated αS. Studies with cultured neurons have observed that overexpression of αS impairs neurotransmitter release (Larsen et al., 1988; Nemani et al., 1988; Lundblad et al., 2010; Phan et al., 2017). Knockout studies in mice showed no early phenotypes, but had earlier onset of neurological issues (Chandra et al., 2004; Wu et al., 2010; Kokhan et al., 2012). This leads to the notion that αS has the potential to be toxic at excessive concentrations, but necessary at normal concentrations for some unknown reasons. Multiple *in vitro* studies have been performed with contradicting observations. Some groups observe that αS inhibits SNARE-dependent membrane fusion (DeWitt and Rhoades, 2013; Lai et al., 2013), while others observed an enhancement (Liu et al., 2004; Lou et al., 2010; Burré et al., 2014). Previously, we demonstrated in an *in vitro* single vesicle-vesicle fusion assay that αS has a very narrow concentration window in which it enhances SNARE-dependent vesicle docking (Lou et al., 2010). Here, in order to better investigate αS’s role on SNARE-dependent membrane fusion, we used a TIRF vesicle-to-supported bilayer merger assay. Unlike the vesicle-to-vesicle platform (Yoon et al., 2006), the flatness of the t-bilayer mimics the target plasma membrane more accurately. This is a necessary improvement to avoid potential artifacts stemming from the αS’s preferential binding to the curved membrane (Varkey et al., 2010; DeWitt and Rhoades, 2013; Snead and Eliezer, 2014). With this assay employing the highly pure protein, we show that αS enhances SNARE-dependent vesicle docking even at excessive concentrations.

Although it may vary widely, the cellular concentration of αS is estimated to be roughly 5 μM (Chung et al., 2002; Seo et al., 2002). Overexpression typically increases αS by a factor of 2 to 3. Thus, our data shows that αS plays a positive role in vesicle-to-bilayer merger under overexpression conditions. Why does overexpression of αS then impair neurotransmitter release? We speculate that soluble aggregates of αS, although small in quantity, interfere with the SNARE function. In fact, it is shown that soluble aggregates of αS can inhibit SNARE-dependent membrane fusion effectively even at a 100 nM monomer concentration (Choi et al., 2013). Overexpression inevitably renders aggregation of αS over time. The small increase of these high molecular weight bands is enough to negate any enhancement by non-aggregated αS, which demonstrates their potency.

Our data shows that the promotive effect of αS on SNARE-dependent bilayer merger is mainly through the enhancement of vesicle docking. Other than the number of vesicle docking, we did not observe significant changes in the merger efficiency, the ratio of instant to delayed merger events, or the kinetics of bilayer merger. However, it has been recently shown that αS plays a role in accelerating fusion pore opening (Logan et al., 2017). Because our assay uses a lipid probe that reports the lipid dynamics, we are not able to test this mechanistic model on fusion pore dynamics, warranting further investigation using the fluorescent content reporters.

**FIGURE 5:**
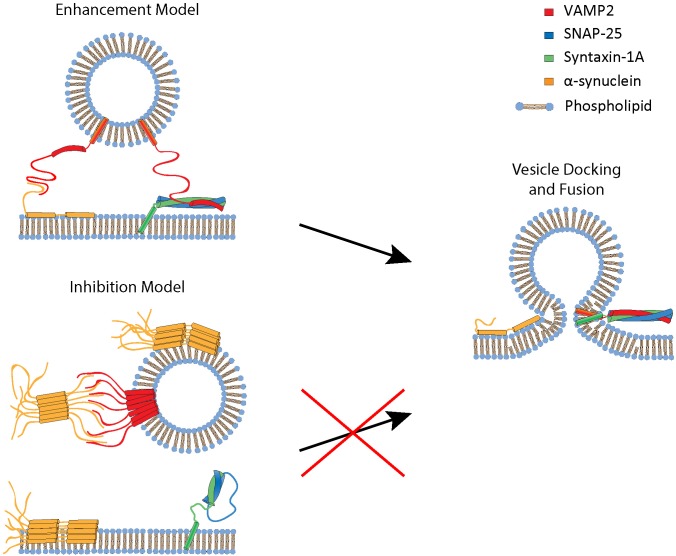
A proposed mechanistic model for enhancement and inhibition of SNARE-dependent vesicle fusion by αS. The enhancement model involves monomeric αS acting as a cross-bridge to facilitate vesicle docking to the phospholipid bilayer. αS has a N-terminal membrane binding region that binds to the phospholipid bilayer and the C-terminal region that interacts with VAMP2 (Lou et al., 2010). The inhibition model involves oligomeric, multivalent αS interacting and clustering VAMP2, thus preventing it from engaging in SNARE complex formation and vesicle fusion.

Combining the present and previous work, two apparent tracks of the regulation of SNARE-dependent membrane fusion by αS emerge (Figure 5). When αS is non-aggregated, it plays a positive role in bilayer merger by promoting vesicle docking. Recently, Lou et al. (2010) proposed a mechanistic model for this positive regulation, envisioning that αS binds to VAMP2 using its unstructured C-terminus. It interacts simultaneously with the target plasma membrane in *trans* using its amphipathic N-terminal region, whereby aiding the recruitment of synaptic vesicles to the plasma membrane. On the other hand, when aggregated, the multivalent oligomeric species could bind to multiple VAMP2 on the vesicle, incapacitating its ability to interact with t-SNARE on the plasma membrane (Choi et al., 2013). As such, the bilayer merger would be severely inhibited.

## Conclusion

Overexpression of αS *in vivo* studies results in diminished neurotransmitter release in cultured neurons. This supports the argument that in addition to the aggregates, excessive amount of non-aggregated αS might be neurotoxic too. Our detailed analysis using *in vitro* single-vesicle bilayer merger assay shows that αS is beneficial for SNARE-dependent vesicle fusion even at excessive concentrations, but the apparent toxicity of αS might be mainly due to the presence of the aggregated species. We observed enhancement of SNARE-dependent vesicle docking and bilayer merger, even in extreme concentrations of pure αS, with 13 fold enhancement at 10 μM αS. The vesicle-to-supported bilayer merger assay could help reveal how SNARE regulators affect each step in SNARE-dependent membrane fusion and thereby advance our understanding of how these regulators affect neurotransmission.

## Data Availability

All datasets generated for this study are included in the manuscript and/or the Supplementary Files.

## Author Contributions

BH and RK contributed equally to the data collection, analysis, and interpretation of the experiments along with the drafting and revising the manuscript. Y-KS was responsible for the directing the research and manuscript drafting.

## Conflict of Interest Statement

The authors declare that the research was conducted in the absence of any commercial or financial relationships that could be construed as a potential conflict of interest.

## Supplementary Material

FIGURE S1 Two round gel filtration of αS. **(A)** Chromatogram from the 1st round of gel filtration. After one round of gel filtration, αS elutes as two overlapping peaks between fractions 7–14. The UV absorbance value has been normalized with respect to the maximum peak intensity. **(B)** SDS-PAGE gel after the 1st round of gel filtration. The fraction numbers indicated on the SDS-PAGE correspond to the fractions shown in part **(A)**. Fractions 7–10 display a high molecular weight bands which correlates to the first peak in the chromatogram. Fractions 11–14, indicated by arrows, were combined, concentrated, and subjected to another round of gel filtration. **(C)** Chromatogram from the 2nd round of gel filtration. After two rounds of gel filtration, αS elutes as a single peak between fractions 10–14. **(D)** SDS-PAGE of the 2nd round of gel filtration. Fractions 12–14 display a molecular weight bands consistent with αS which correlates to the single peak in the chromatogram. A conservative approach was used and fractions containing an arrow underneath were combined, concentrated and used for TIRF experiments.

FIGURE S2 SDS-PAGE of purified αS before and after storage at -80°C. **(A)** SDS-PAGE gel of αS immediately after two rounds of gel filtration before storing at -80°C. **(B)** SDS-PAGE gel of the same αS after 2 weeks of storage at -80°C.
