# Supplementary figures and images for: Alpha-Synuclein Continues to Enhance SNARE-Dependent Vesicle Docking at Exorbitant Concentrations
Source: Front Neurosci. 2019 Mar 21;13:216. doi: 10.3389/fnins.2019.00216 (PMC6437117; doi:10.3389/fnins.2019.00216)

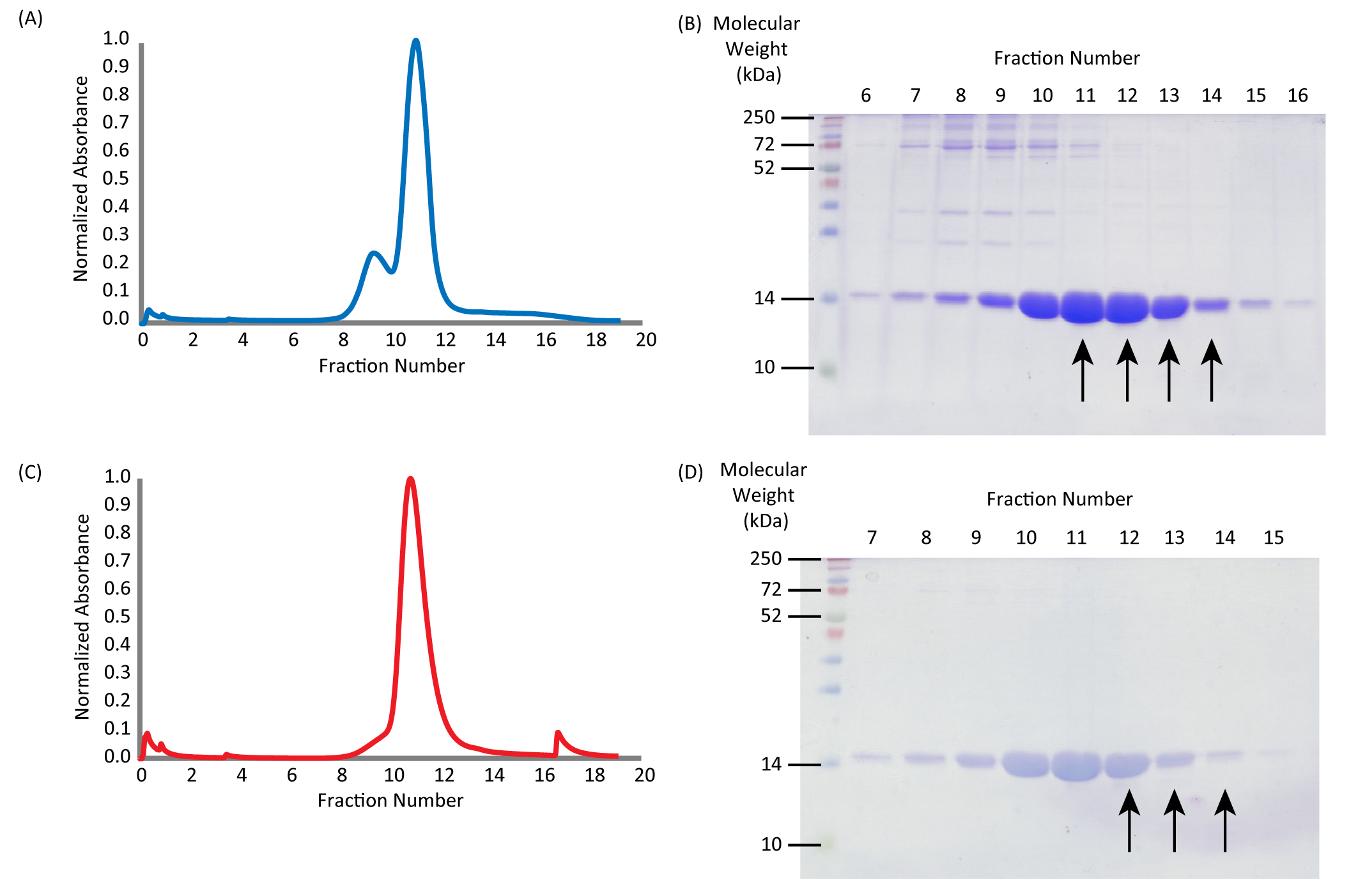

Supplement: FIGURE S1 — Two round gel filtration of αS. (A) Chromatogram from the 1st round of gel filtration. After one round of gel filtration, αS elutes as two overlapping peaks between fractions 7–14. The UV absorbance value has been normalized with respect to the maximum peak intensity. (B) SDS-PAGE gel after the 1st round of gel filtration. The fraction numbers indicated on the SDS-PAGE correspond to the fractions shown in part (A). Fractions 7–10 display a high molecular weight bands which correlates to the first peak in the chromatogram. Fractions 11–14, indicated by arrows, were combined, concentrated, and subjected to another round of gel filtration. (C) Chromatogram from the 2nd round of gel filtration. After two rounds of gel filtration, αS elutes as a single peak between fractions 10–14. (D) SDS-PAGE of the 2nd round of gel filtration. Fractions 12–14 display a molecular weight bands consistent with αS which correlates to the single peak in the chromatogram. A conservative approach was used and fractions containing an arrow underneath were combined, concentrated and used for TIRF experiments. [file Image_1.TIF]

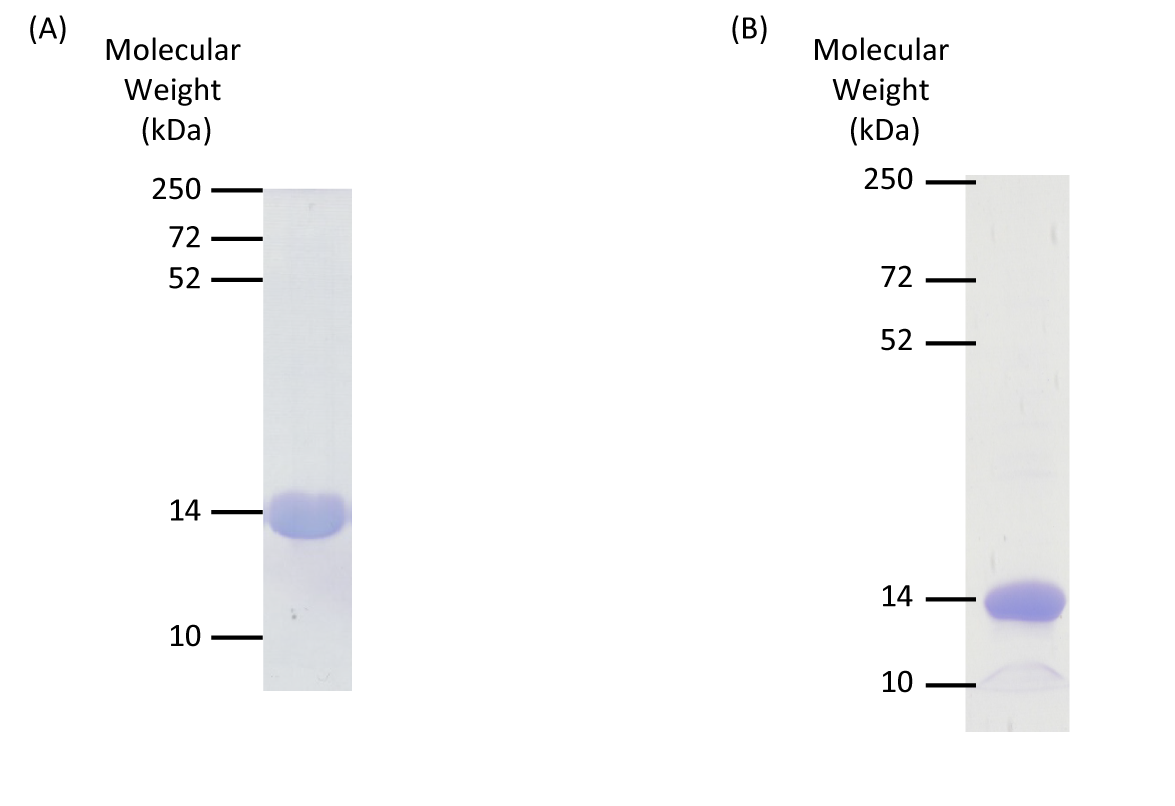

Supplement: FIGURE S2 — SDS-PAGE of purified αS before and after storage at -80°C. (A) SDS-PAGE gel of αS immediately after two rounds of gel filtration before storing at -80°C. (B) SDS-PAGE gel of the same αS after 2 weeks of storage at -80°C. [file Image_2.TIF]
